# Field Implementation of Buserelin-Supplemented Boar Semen Protocol at the First Insemination: Farrowing Rate and Litter Size in Gilts and Sows Under Tropical Conditions

**DOI:** 10.3390/ani16193101

**Published:** 2026-10-02

**Authors:** Preechaphon Taechamaeteekul, Pornpavee Athsukitkul, Kita Wongvekin, Jittichai U-Thaichan, Yanthicha Boonmee, Tip-apa Akkhaphan, Pisit Sirithanyakul, Rafa Boonprakob, Hongyao Lin, Miquel Collell, Padet Tummaruk

**Affiliations:** 1 Department of Obstetrics, Gynaecology and Reproduction, Faculty of Veterinary Science, Chulalongkorn University, Bangkok 10330, Thailand; taechamaeteekul.p@gmail.com (P.T.); tipapa.a1999@gmail.com (T.-a.A.); 2 Faculty of Veterinary Science, Chulalongkorn University, Bangkok 10330, Thailand; ppv.ath@gmail.com (P.A.); kitawongvekin3@gmail.com (K.W.); jittichai.uth@gmail.com (J.U.-T.); yanthichaboonmee@gmail.com (Y.B.); 3 Intervet (Thailand) Co., Ltd., Bangkok 10330, Thailand; pisit.sirithanyakul@msd.com; 4 Livestock Research, Department of Quality Assurance and Animal Health Office, Livestock Production, Betagro Group, Bangkok 10210, Thailand; rafab@betagro.com; 5 Merck Animal Health, 2 Giralda Farms, Madison, NJ 07940, USA; hongyao.lin@msd.com (H.L.); miquel.collell@merck.com (M.C.); 6 Centre of Excellence in Swine Reproduction, Chulalongkorn University, Bangkok 10330, Thailand; 7 The Academy of Science, The Royal Society of Thailand, Bangkok 10210, Thailand

**Keywords:** artificial insemination, buserelin, farrowing rate, swine, tropical climate

## Abstract

Maximizing farrowing rate and litter size is essential for improving reproductive efficiency and profitability in swine production. Both outcomes depend on the timing of artificial insemination (AI) relative to ovulation; however, predicting ovulation is challenging because estrus duration and the interval from estrus onset to ovulation vary considerably among females. Therefore, this study aimed to determine whether supplementing extended boar semen with 4–8 µg of buserelin acetate at the first insemination improves the reproductive performance of gilts and sows under tropical commercial conditions. A randomized controlled field trial was conducted involving 2780 Landrace × Yorkshire females across five commercial herds in tropical regions (herds A–E). The females (parity 3.0 ± 2.1) comprised gilts (*n* = 412), sows (*n* = 2280), and repeat-service females (*n* = 88). They were randomly assigned to receive either standard extended semen (control; *n* = 1438) or extended semen supplemented with 4 μg (1.0 mL; *n* = 121), 5 μg (1.25 mL; *n* = 1052), or 8 μg (2.0 mL; *n* = 169) of buserelin acetate per 90 mL insemination dose (treatment; *n* = 1342). The overall farrowing rate was 88.5% and ranged from 84.5% to 90.0% among herds. Buserelin supplementation increased the farrowing rate compared with the control (90.1% vs. 86.9%, *p* = 0.009), representing an improvement of 3.2 percentage points. In Herd C, supplementation increased the farrowing rate from 86.5% to 90.1% (*p* = 0.036). The overall total number of piglets born was 14.1 ± 3.2 per litter. Litter size did not differ significantly between groups in any herd (all *p* > 0.05), although buserelin-treated females generally had numerically more total-born and live-born piglets. Among gilts, buserelin tended to increase the total number born (12.7 ± 0.4 vs. 11.8 ± 0.4 piglets, *p* = 0.083), but not the number born alive (11.3 ± 0.4 vs. 10.6 ± 0.4 piglets, *p* = 0.258). No significant effects were observed in sows for total-born or live-born piglets (+0.3 and +0.4 piglets, *p* = 0.380 and 0.376, respectively), or in repeat-service females (−0.4 and +0.2 piglets, *p* = 0.735 and 0.883, respectively). These findings indicate that supplementing extended semen with buserelin acetate at the first insemination is a practical, non-invasive strategy for improving farrowing success in commercial swine herds under tropical conditions. Further studies should clarify the underlying endocrine and reproductive mechanisms and identify the physiological or reproductive characteristics of females most likely to benefit from additional hormonal stimulation.

## Simple Summary

In swine production, successful pregnancy depends on several factors, including the timely presence of sperm in the reproductive tract, ideally within 24 h before ovulation. However, the timing of ovulation varies among females and may be further affected by hot weather. Farmers therefore commonly inseminate gilts and sows two or three times during standing estrus. Buserelin is a hormone used to induce and synchronize ovulation. Although usually administered by injection, it can be added directly to semen, avoiding the additional handling and stress associated with injection. This study evaluated 2780 gilts and sows from five commercial herds under tropical conditions. Control females received conventional semen during two or three inseminations, whereas treated females received two inseminations, with buserelin-supplemented semen used only for the first insemination. Treated females had a higher farrowing rate than control females (90.1% vs. 86.9%, *p* = 0.009). Litter size did not differ significantly between groups, although treated gilts tended to produce more piglets (+0.9 piglets per litter, *p* = 0.083). These findings suggest that adding buserelin to semen may offer a simple method for improving farrowing success in commercial swine herds without an additional hormone injection.

## 1. Introduction

In swine production, maximizing litter size and farrowing rate is critical for improving reproductive efficiency and economic returns [1,2]. These reproductive outcomes are highly dependent on the accurate timing of artificial insemination (AI) relative to ovulation [3]. However, predicting ovulation remains challenging in both gilts and sows because of prolonged estrus and substantial individual variation in the interval from estrus onset to ovulation [4,5]. This challenge may be particularly important under tropical conditions, where ovulation has been reported to occur at 64.3 ± 19.3 h [4] and 63.6 ± 9.5 h [6] after the onset of estrus (mean ± SD). Consequently, multiple inseminations are often performed during standing estrus to ensure that viable sperm are present at the time of ovulation. Nevertheless, farrowing rates and litter sizes may remain suboptimal, particularly during the hot season [7,8]. Therefore, strategies to control the timing of ovulation in specific groups of females, herds, or seasons may help maintain reproductive performance under tropical conditions.

Fixed-time artificial insemination (FTAI) protocols have been developed to synchronize ovulation, thereby allowing AI to be performed at a predetermined time without the need for continuous estrus detection [4,9,10]. These protocols commonly use equine chorionic gonadotropin (eCG) to stimulate follicular development, followed by a gonadotropin-releasing hormone (GnRH) analog, such as buserelin or triptorelin, to induce a luteinizing hormone (LH) surge and to trigger ovulation approximately 36–38 h after administration. FTAI may be particularly advantageous in animals with variable estrus expression and under tropical conditions, where timely estrus detection can be challenging [9]. By reducing the number of inseminations and coordinating the timing of insemination with ovulation, FTAI can improve reproductive efficiency and simplify labor management in commercial swine operations.

To control the timing of ovulation, exogenous GnRH is typically administered at the onset of standing estrus or when ovarian follicles reach ≥6.0 mm in diameter [11]. A single intramuscular injection of 10 µg of GnRH effectively induces an LH surge, resulting in ovulation approximately 37.5 ± 3.2 h later [6] (mean ± SD). This approach facilitates the implementation of FTAI protocols by improving ovulation synchronization and reproductive outcomes [9,12]. In addition to their use in ovulation synchronization, GnRH analogs have been investigated for their potential fertility-enhancing effects when added directly to extended semen. Previous studies have demonstrated that supplementing boar semen with 2–10 µg of buserelin acetate per dose at the first insemination significantly increases farrowing rate [13] and litter size [14,15]. Compared with intramuscular administration, this approach is less invasive and more practical for on-farm application. However, these findings were based on a limited number of animals and therefore require validation in larger commercial populations [14,15]. Accordingly, the present study evaluated the effects of supplementing extended boar semen with buserelin acetate at the first insemination on farrowing rate and litter size in gilts and sows under tropical commercial conditions. Reproductive outcomes were also compared among herds to identify factors that may influence the effectiveness of buserelin supplementation.

## 2. Materials and Methods

### 2.1. Animals and Study Design

This study was conducted in accordance with the ethical principles and guidelines for animal research established by the National Research Council of Thailand (NRCT). The experimental protocol was approved by the Chulalongkorn University Institutional Animal Care and Use Committee (IACUC; protocol no. 2531067) and complied with relevant university and Thai government regulations governing the care and use of animals in research.

A randomized controlled field trial was conducted from May 2024 to July 2025 involving 2780 Landrace × Yorkshire crossbred females from five commercial pig herds in tropical regions: A (*n* = 748), B (*n* = 163), C (*n* = 1419), D (*n* = 80), and E (*n* = 370). During the study period, monthly mean regional ambient temperatures ranged from 29.5 to 32.1 °C in the hot season, 28.8 to 30.2 °C in the rainy season, and 24.7 to 28.1 °C in the cool season. All females were clinically healthy, exhibited normal estrous cycles, and were managed according to each herd’s standard practices. At insemination, mean (±SD) parity was 3.0 ± 2.1 (range, 0–12). Based on reproductive status, females were classified as gilts (*n* = 412), sows (*n* = 2280), or repeat-service females (*n* = 88). The repeat-service group comprised seven gilts and 81 sows of parities 1–6. Mean lactation length was 24.0 ± 2.0 days (mean ± SD). Within each herd, females were randomly assigned in approximately equal numbers to the control and treatment groups within each batch and insemination day. The herd manager performed the allocation by balancing the control and treatment groups within each parity group. Treatment and insemination personnel were not blinded because they were responsible for administering buserelin during insemination.

This study evaluated the effects of supplementing extended Duroc boar semen with buserelin acetate on the reproductive performance of gilts and sows. All Duroc boars were proven sires aged 1–3 years and were routinely used for artificial insemination in their respective herds. Within each herd, semen from each boar was used in both the control and treatment groups. Neither boars nor their semen were shared across herds for biosecurity reasons. Females were randomly assigned to a control group (*n* = 1438), inseminated with standard extended boar semen, or a treatment group (*n* = 1342), inseminated with extended semen supplemented with buserelin acetate only at the first insemination. Treated females were classified into three subgroups according to the amount of buserelin acetate added per 90 mL insemination dose: 4 µg (*n* = 121), 5 µg (*n* = 1052), or 8 µg (*n* = 169). Treatment doses varied by herd: herd A used 4 and 8 µg (*n* = 121 and 169, respectively), whereas herds B–E used 5 µg (*n* = 1052). This dose range was selected based on previous studies using 2–10 µg per insemination dose [13,14,15]. Buserelin acetate (4 µg/mL; Receptal^®^, Merck Animal Health, Rahway, NJ, USA) was added to the first 90 mL insemination dose immediately before the insemination. Volumes of 1.0, 1.25, or 2.0 mL were added to provide 4, 5, or 8 µg of buserelin acetate, respectively.

### 2.2. Insemination Protocol

Artificial insemination (AI) protocols varied among herds but followed common timing criteria. The first insemination was performed 8–12 h after the onset of standing estrus in sows and at its onset in gilts and repeat-breeding sows. Subsequent inseminations were performed at 8–12 h intervals while females remained in standing estrus. Control females received two or three inseminations, depending on the duration of the standing response, whereas treated females received two. In the treatment groups, buserelin-supplemented semen was used only for the first insemination; the second was performed approximately 12 h later using standard extended semen without buserelin. The two-insemination regimen was selected based on the anticipated induction of ovulation within 36–40 h after buserelin administration and intended to avoid insemination after ovulation.

Semen samples with subjectively assessed sperm motility >80% were selected for AI. Sperm concentration was measured using a spectrophotometer (SpermaCue^®^, Minitüb GmbH, Tiefenbach, Germany). Ejaculates were then diluted to provide 3.0 × 10^9^ spermatozoa per 90 mL insemination dose using M III^®^ (Minitübe International AG, Tiefenbach, Germany) in all herds except herd B, which used a commercial short-term extender, AI expert^®^ (Green Innovative Biotechnology, Bangkok, Thailand). Semen volume and sperm concentration were standardized across herds. Extended semen was stored at 17 °C and used within 24 h. Gilts received cervical insemination using a standard foam-tip AI catheter, whereas sows received post-cervical insemination. All inseminations were performed by trained herd personnel according to their respective herds’ standard procedures.

### 2.3. Data Collection

Each female was identified using an individual record card placed above her crate. At enrollment, the assigned treatment code was recorded on the card; however, herd personnel responsible for animal care and data collection were blinded to the corresponding treatment. Recorded information included female identification, reproductive status (gilt or sow), parity, insemination date, and expected farrowing date. At farrowing, the farrowing date and the numbers of piglets born alive (BA), stillborn piglets (SB), and mummified fetuses (MF) per litter were recorded. Total piglets born (TB) was calculated as the sum of BA, SB, and MF.

### 2.4. Statistical Analysis

All statistical analyses were performed using SAS version 9.4 (SAS Institute Inc., Cary, NC, USA). Descriptive statistics for quantitative variables, including litter traits, were calculated using the MEANS procedure and are presented as means ± SD and ranges. Categorical variables, including farrowing rate, were analyzed using the FREQ procedure and are presented as percentages. Differences in farrowing rates between groups were evaluated using the chi-square test. In addition, factors associated with farrowing rate were evaluated simultaneously using a generalized linear mixed model fitted with the GLIMMIX procedure of SAS. The model included treatment group (control or buserelin), female status (gilt, sow, or repeat-service female), mating season (hot, rainy, or cool), herd (A–E), and two-way interactions, including the interaction between treatment group and female status. Two-way interactions were assessed for statistical significance in a stepwise manner, and only significant interactions were retained in the final model. Farrowing outcome was modeled using a binomial distribution with a logit link function. Least squares means were estimated using the LSMEANS statement and compared using the least significant difference test. Model-adjusted results are presented as least-squares means ± SEM.

Litter traits, including the total number of piglets born, number born alive, stillbirth percentage, and mummified fetus percentage, were analyzed using the general linear model (GLM) procedure. The model included treatment group (control or buserelin), female status (gilt, sow, or repeat-service female), mating season (hot, rainy, or cool), herd (A–E), and the two-way interactions between treatment group and female status, mating season and herd. Least-squares means were estimated using the LSMEANS statement and compared using the least significant difference test. Results are presented as least-squares means ± SEM.

The normality of model residuals was assessed using the Shapiro–Wilk test in the UNIVARIATE procedure and by examining normal probability plots, skewness, and kurtosis. Differences were considered statistically significant at *p* < 0.05.

## 3. Results

### 3.1. Descriptive Statistics

**Figure 1:**
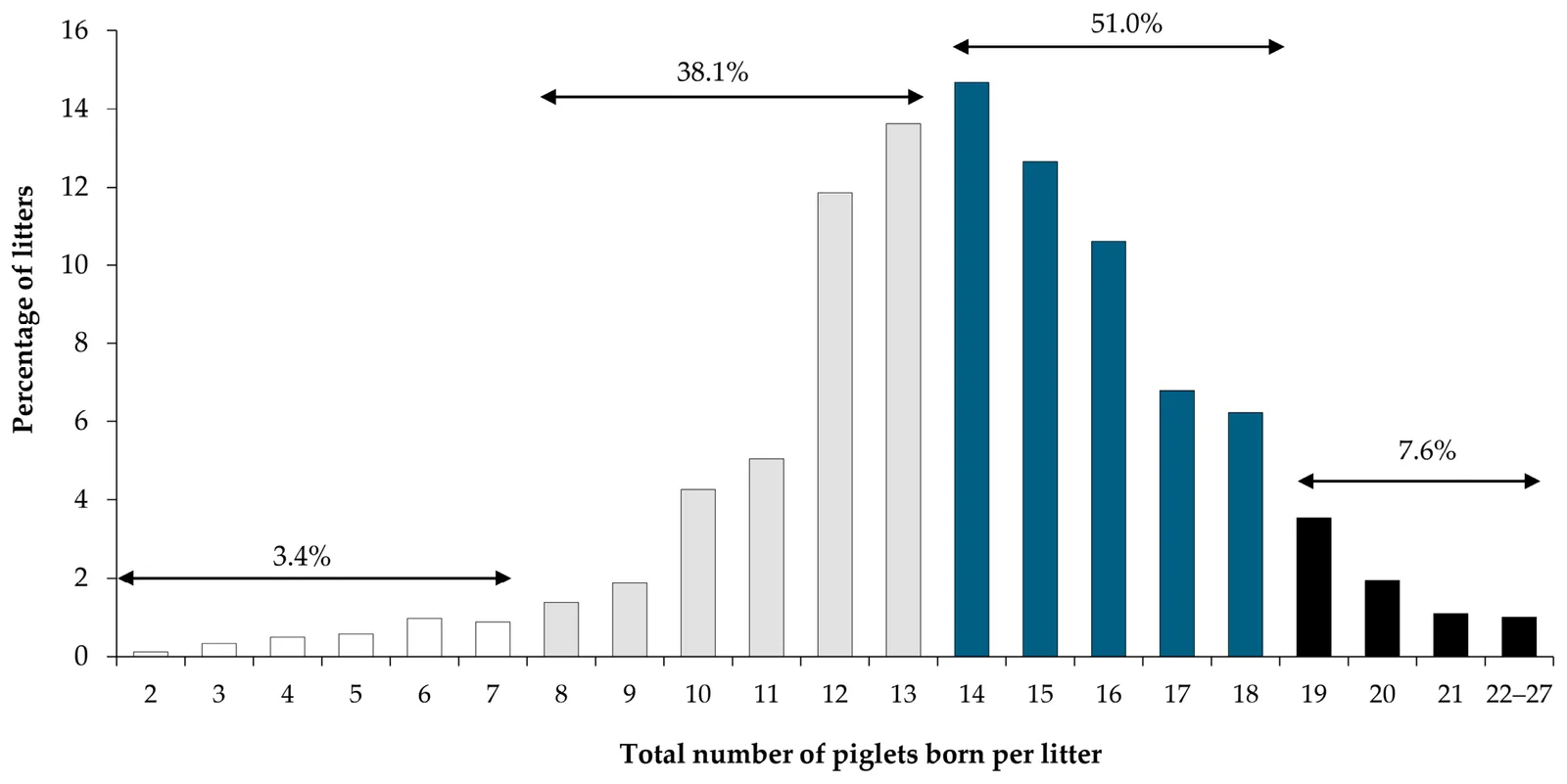
Frequency distribution of total litter sizes among 2459 Landrace × Yorkshire crossbred females from five commercial herds under tropical conditions. Litters were classified as ≤7 piglets (white bars; 3.4%), 8–13 piglets (light-gray bars; 38.1%), 14–18 piglets (blue bars; 51.0%), or ≥19 piglets (black bars; 7.6%).

Table 1 summarizes the overall reproductive performance of the females included in the study. Across the five commercial herds under tropical conditions, 2459 of the 2780 inseminated females farrowed, resulting in an overall farrowing rate of 88.5 %. Farrowing rates varied among herds, ranging from 84.5% to 90.0%. Mean parity was 3.0 ± 2.1 at insemination and 4.0 ± 2.1 at farrowing. Across all herds, the mean total number of piglets born was 14.1 ± 3.2 per litter, of which 12.9 ± 3.4 were born alive. The distribution of litter size at birth is presented in Figure 1. Overall, 3.4% of females produced ≤7 piglets, 38.1% produced 8–13 piglets, and 58.5% produced ≥14 piglets per litter. The overall percentages of stillborn piglets and mummified fetuses were 4.7% and 3.6%, respectively.

Reproductive performance varied descriptively among the five commercial herds (Table 2). Females in Herd B had the lowest mean parity at service and farrowing, whereas those in Herd D had the highest. Farrowing rates were generally comparable across herds but were numerically highest in Herds A and D and lowest in Herd B. Herd A had the largest total-born and live-born litter sizes but also the highest stillbirth percentage. By contrast, Herd E had the lowest stillbirth percentage. The percentage of mummified fetuses was highest in Herd B and lowest in Herd A.

### 3.2. Effect of Buserelin Supplementation on Farrowing Rate

**Figure 2:**
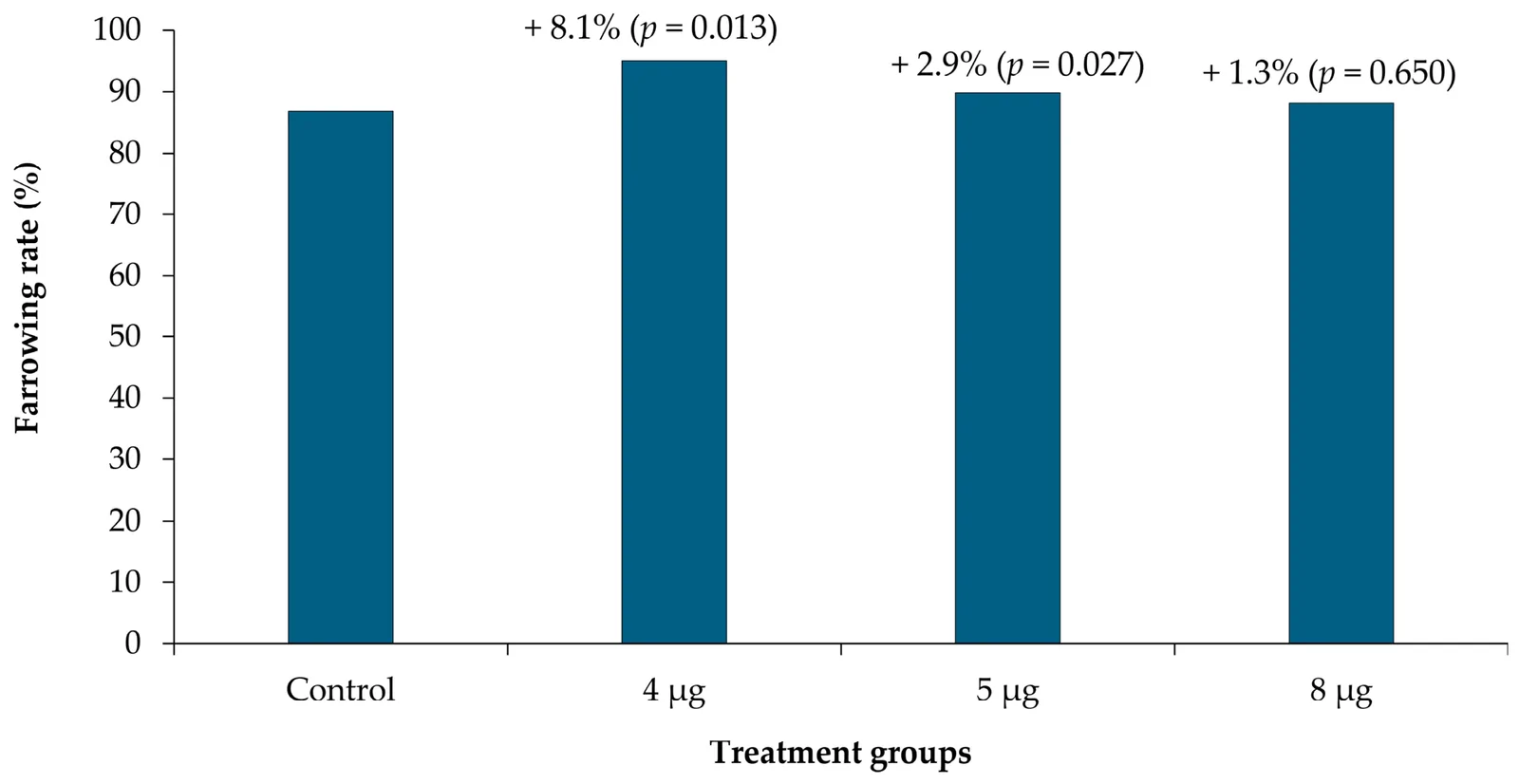
Farrowing rates of females inseminated with conventional extended semen without buserelin (control) or with semen supplemented with 4, 5, or 8 µg of buserelin at the first artificial insemination, across five commercial herds.

Across all herds, supplementation of extended boar semen with buserelin significantly increased the farrowing rate from 86.9% in the control group to 90.1% in the treatment group (*p* = 0.009). Figure 2 compares farrowing rates among females receiving conventional extended semen or semen supplemented with 4, 5, or 8 µg of buserelin at the first artificial insemination across five commercial herds. Compared with the control group, farrowing rates were significantly higher with 4 µg (*p* = 0.013) and 5 µg (*p* = 0.027) of buserelin, whereas no significant difference was observed with 8 µg (*p* = 0.650).

**Figure 3:**
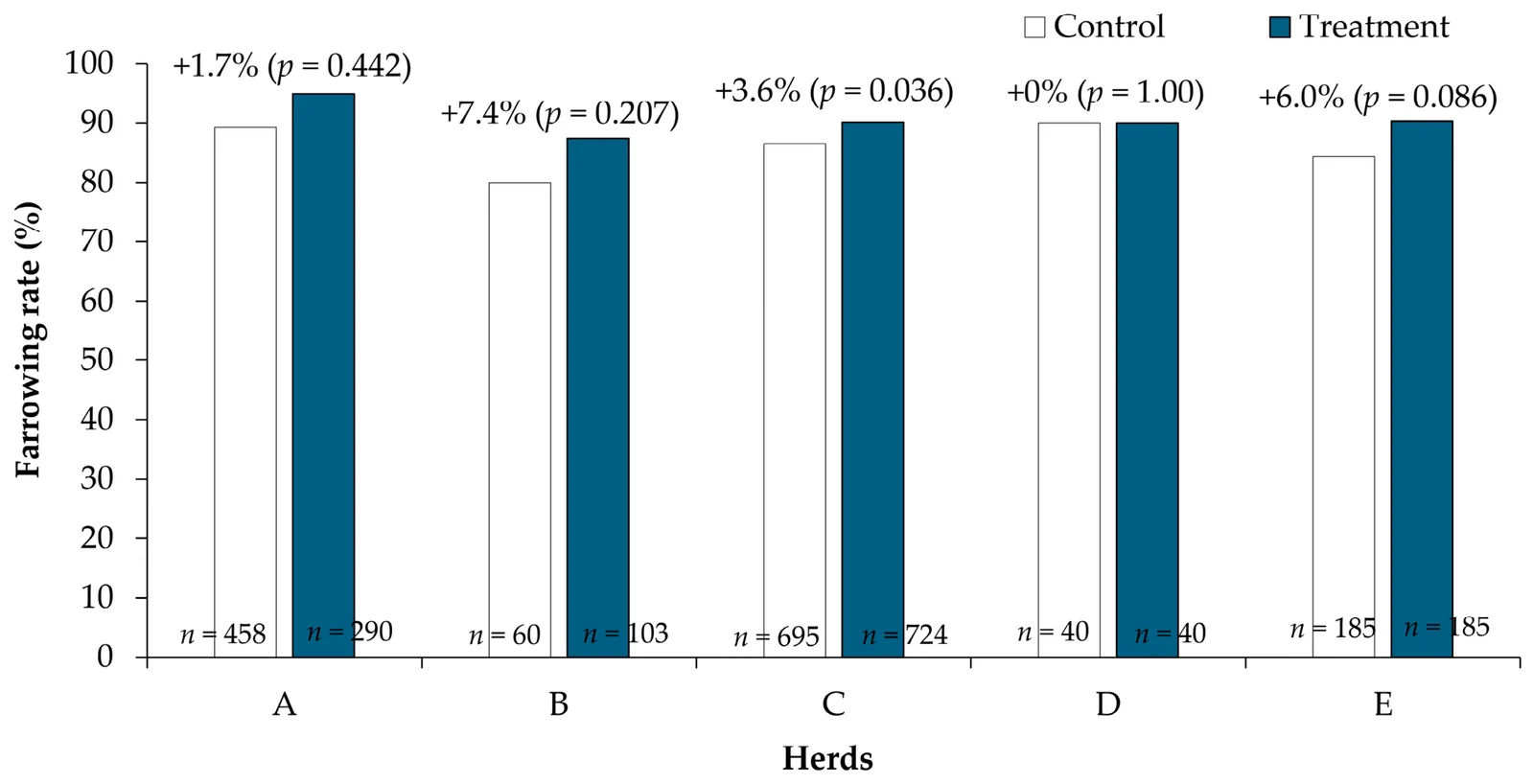
Farrowing rates by herd of females inseminated at the first artificial insemination with conventional extended semen without buserelin (control) and with semen supplemented with 4 or 8 µg of buserelin in herd A or 5 µg in herds B–E (treatment).

Herd-specific analyses showed variation in the response to buserelin supplementation (Figure 3). Farrowing rate increased significantly in Herd C (*p* = 0.036) and tended to improve in Herd E (*p* = 0.086). In Herds A and B, farrowing rates were numerically higher in the buserelin groups than in the control groups by 1.7 and 7.4 percentage points, respectively, although these differences were not statistically significant. No difference between treatment groups was observed in Herd D.

**Figure 4:**
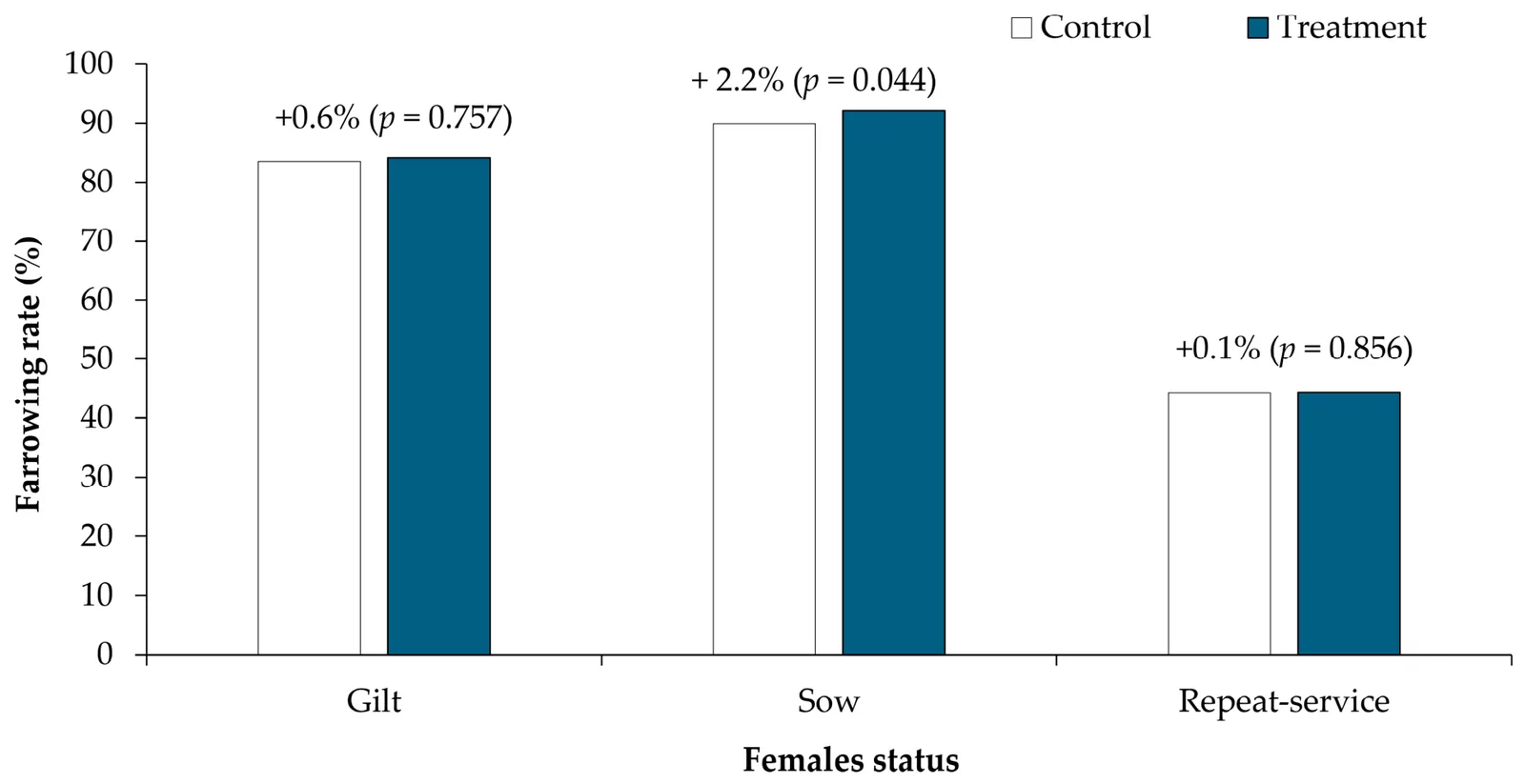
Farrowing rates of gilts, sows, and repeat-service females in the control and buserelin-treatment groups. Values above the bars indicate the percentage-point difference between the treatment and control groups and the corresponding *p* value within each female-status category. Supplementation of boar semen with buserelin at the first insemination increased the farrowing rate in sows (*p* = 0.044), whereas no significant differences were observed in gilts or repeat-service females (*p* > 0.05).

When treatment (buserelin supplementation of extended boar semen), herd, season, and female status (gilt, sow, or repeat-service female) were included simultaneously in the model, the main effects of treatment (*p* = 0.595), herd (*p* = 0.698), and season (*p* = 0.305) on farrowing rate were not significant, whereas the effect of female status was significant. Sows had a higher farrowing rate (91.0%) than gilts (83.7%, *p* < 0.001) and repeat-service females (44.3%, *p* < 0.001). Examination of the treatment-by-female-status interaction showed that buserelin supplementation significantly increased the farrowing rate in sows from 89.9% to 92.1%, an increase of 2.2 percentage points (*p* = 0.044). No significant improvement was observed in gilts or repeat-service females (Figure 4).

### 3.3. Effect of Buserelin Supplementation on Litter Size

The total number of piglets born per litter (13.1 ± 0.4 vs. 13.4 ± 0.4, *p* = 0.592) and the number born alive (11.7 ± 0.4 vs. 12.1 ± 0.4, *p* = 0.493) did not differ significantly between the control and buserelin-treated groups, although the treatment group showed numerical increases of 0.3 and 0.4 piglets per litter, respectively. Similarly, the percentages of stillborn piglets (5.2 ± 1.1 vs. 4.4 ± 1.2, *p* = 0.624) and mummified fetuses (5.3 ± 1.4 vs. 4.6 ± 1.5, *p* = 0.624) per litter did not differ significantly between groups.

**Figure 5:**
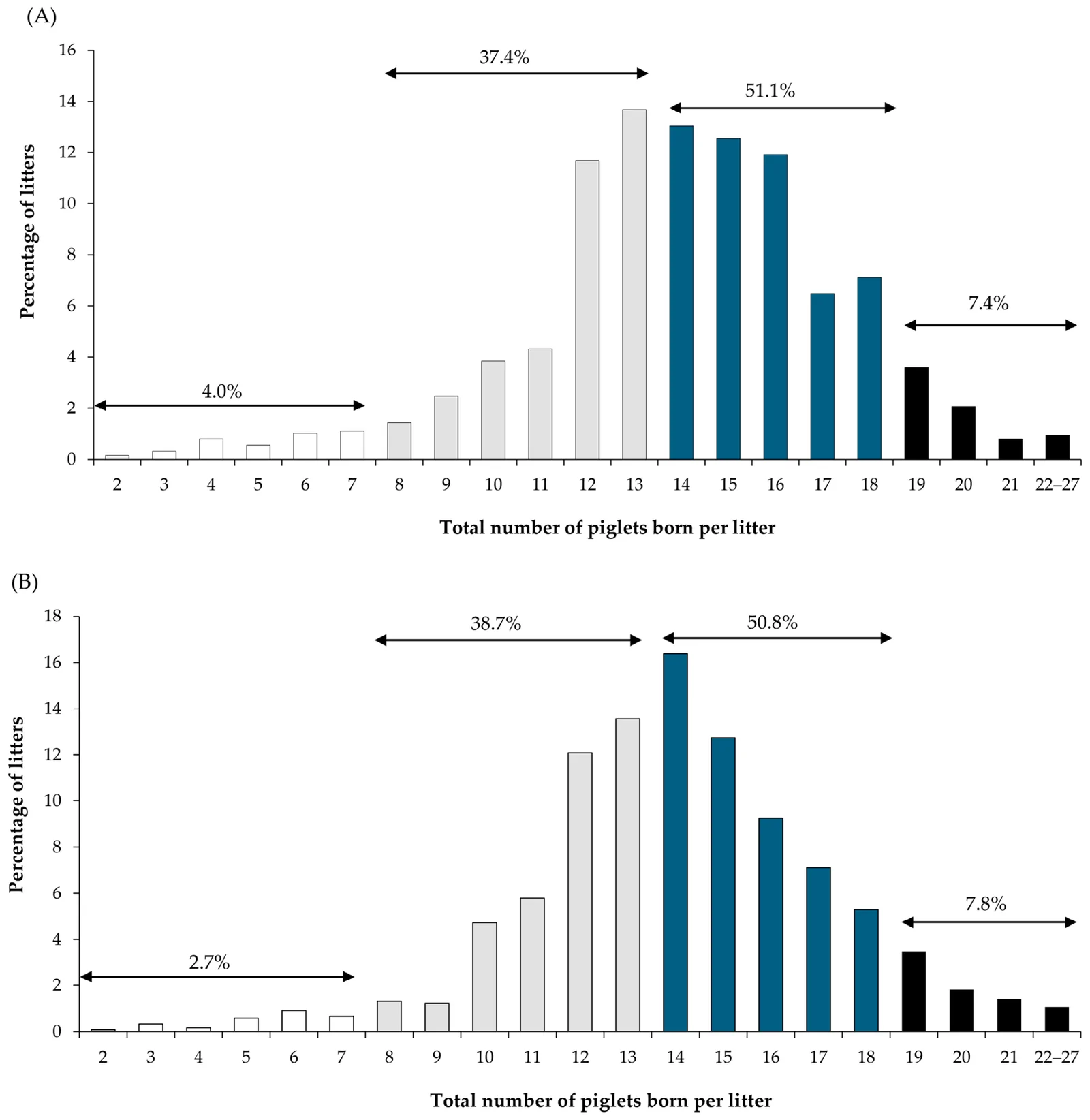
Frequency distributions of total litter size among Landrace × Yorkshire crossbred females inseminated with (**A**) conventional extended semen without buserelin (control) and (**B**) extended semen supplemented with 4–8 µg of buserelin at the first artificial insemination. Litters were classified as ≤7 piglets (white bars), 8–13 piglets (light-gray bars), 14–18 piglets (blue bars), or ≥19 piglets (black bars).

The frequency distributions of total litter size in the control and buserelin-treated groups are presented in Figure 5A and Figure 5B, respectively. Overall, the distributions were broadly similar between groups. Compared with the control group, the buserelin-treated group had a lower proportion of litters with ≤7 piglets (2.7% vs. 4.0%), slightly higher proportions with 8–13 piglets (38.7% vs. 37.4%) and ≥19 piglets (7.8% vs. 7.4%), and a comparable proportion with 14–18 piglets (50.8% vs. 51.1%). The most frequently observed litter size was 13 piglets in the control group and 14 piglets in the buserelin-treated group.

**Figure 6:**
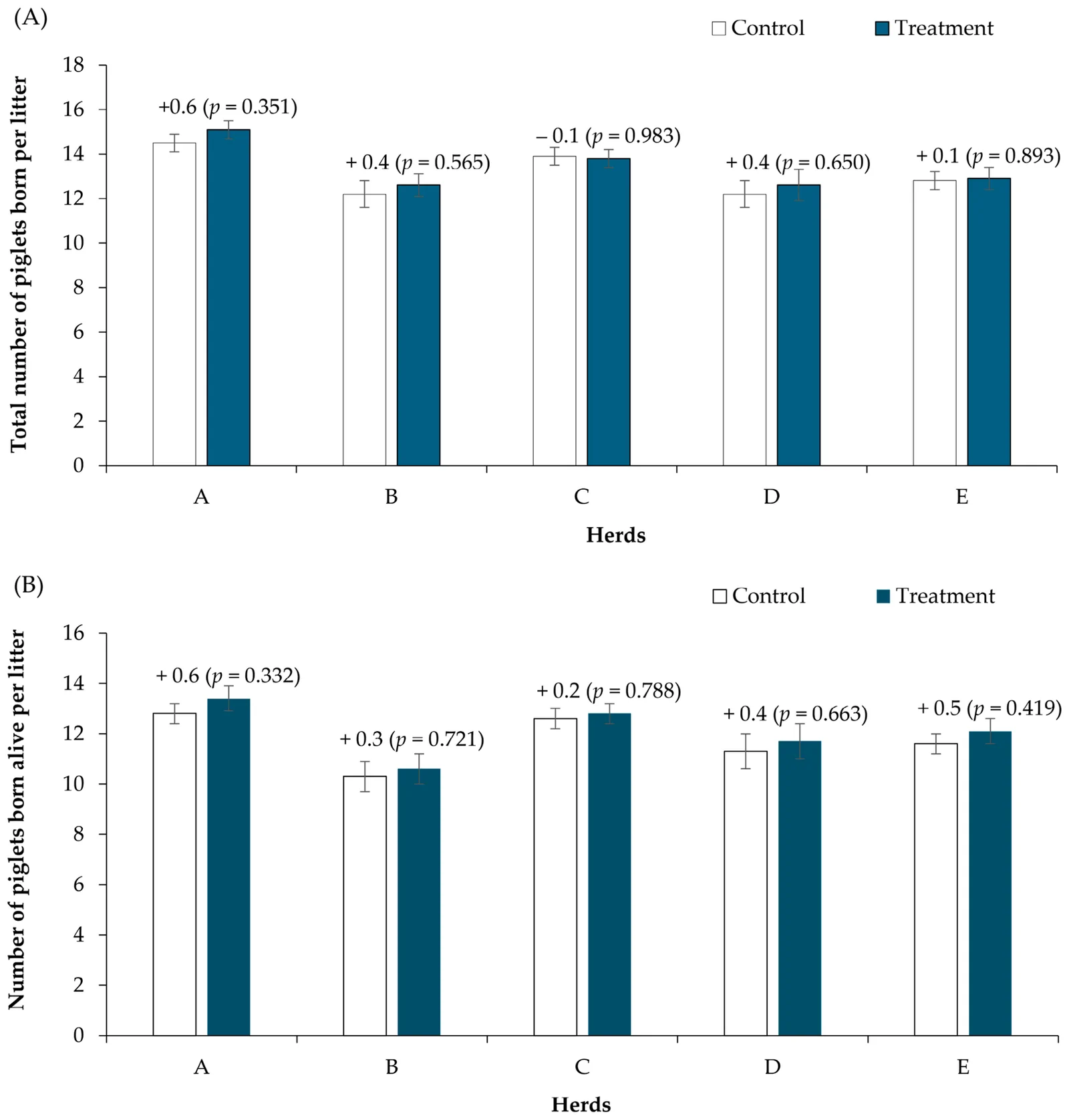
Total number of piglets born per litter (**A**) and number of piglets born alive per litter (**B**) in Landrace × Yorkshire crossbred females inseminated with conventional extended semen without buserelin (control; white bars) or semen supplemented with 4–8 µg of buserelin at the first artificial insemination (treatment; blue bars) across Herds A–E. Data are presented as least-squares means ± SEM. Values above each pair of bars indicate the difference between the treatment and control groups and the corresponding *p* value.

The effects of buserelin supplementation on litter traits across the five herds are presented in Figure 6. The total number of piglets born did not differ significantly between the treatment and control groups in any herd (all *p* > 0.05, Figure 6A). Nevertheless, buserelin-treated females had numerically larger total litter sizes in Herds A, B, D, and E, with increases ranging from 0.1 to 0.6 piglets per litter, whereas a negligible decrease of 0.1 piglets per litter was observed in Herd C. Similarly, the number of piglets born alive did not differ significantly between groups in any herd (all *p* > 0.05, Figure 6B). However, buserelin-treated females consistently produced numerically more live-born piglets across all herds, with increases ranging from 0.2 to 0.6 piglets per litter.

**Figure 7:**
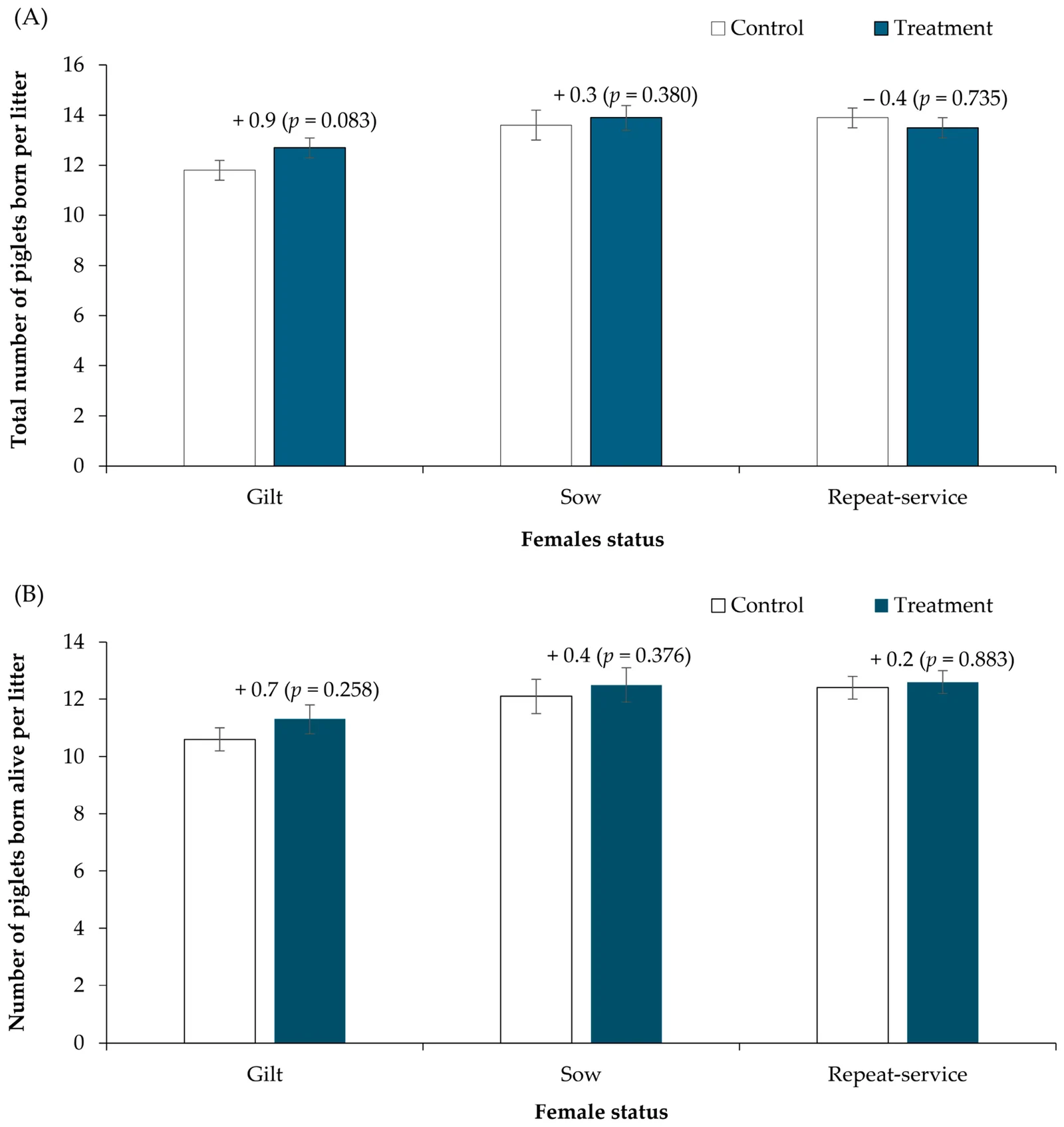
Total number of piglets born per litter (**A**) and number of piglets born alive per litter (**B**) in control and buserelin-treated females, according to reproductive status (gilts, sows, and repeat-service females). Bars represent least-squares means ± SEM. Values above the bars indicate the difference between the treatment and control groups and the corresponding *p* value within each reproductive-status category.

The effects of treatment on litter traits according to female reproductive status are shown in Figure 7. Among gilts, the treatment group tended to have a greater total number of piglets born per litter than the control group (12.7 ± 0.4 vs. 11.8 ± 0.4 piglets; difference, +0.9 piglets, *p* = 0.083). However, the number of piglets born alive did not differ significantly between groups (11.3 ± 0.4 vs. 10.6 ± 0.4 piglets, difference, +0.7 piglets, *p* = 0.258). Among sows, treatment increased the total number born and the number born alive by 0.3 and 0.4 piglets per litter, respectively, although neither difference was significant (*p* = 0.380 and *p* = 0.376, respectively). Similarly, no treatment effects were observed in repeat-service females for either the total number born (difference, −0.4 piglets, *p* = 0.735) or the number born alive (difference, +0.2 piglets, *p* = 0.883).

**Figure 8:**
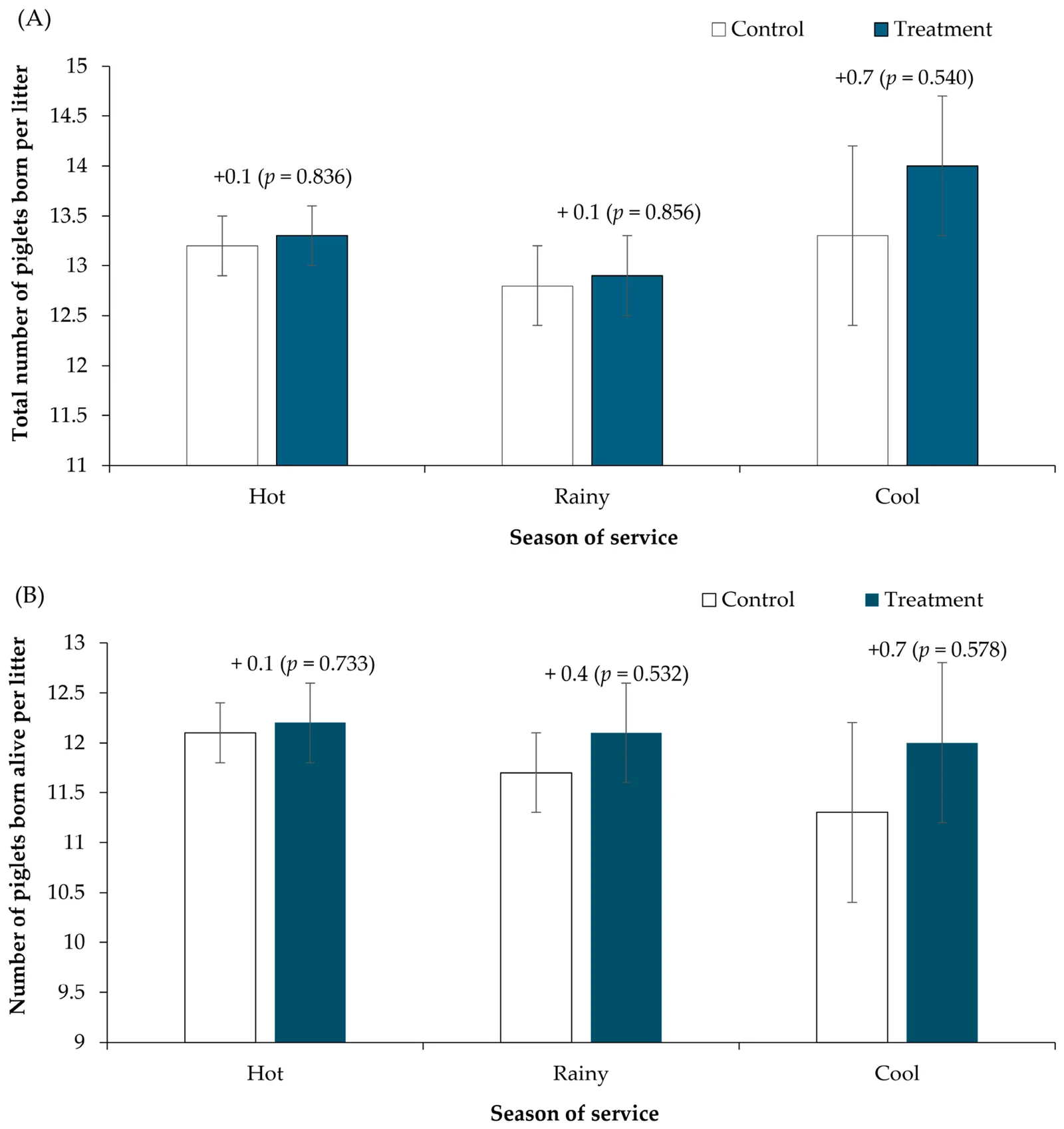
Total number of piglets born per litter (**A**) and number of piglets born alive per litter (**B**) in the control and buserelin-treatment groups according to season of service. Bars represent least-squares means ± SEM. Values above the bars indicate the difference between the treatment and control groups and the corresponding *p* value within each season.

The effects of buserelin treatment on litter traits according to season of service are shown in Figure 8. The treatment group consistently had numerically higher total numbers of piglets born and born alive than the control group across all seasons. However, the total number born did not differ significantly between groups during the hot (+0.1 piglets, *p* = 0.836), rainy (+0.1 piglets, *p* = 0.856), or cool season (+0.7 piglets, *p* = 0.540) (Figure 8A). Similarly, the number of piglets born alive was numerically higher in the treatment group during the hot (+0.1 piglets, *p* = 0.733), rainy (+0.4 piglets, *p* = 0.532), and cool seasons (+0.7 piglets, *p* = 0.578), although none of these differences was statistically significant (Figure 8B).

### 3.4. Effect of Buserelin Dose

The effects of buserelin dose on farrowing rate are presented in Figure 2. Overall, supplementation of the first AI semen dose with 4 µg or 5 µg of buserelin increased farrowing rate by 8.1 percentage points (*p* = 0.013) and 2.9 percentage points (*p* = 0.027), respectively, compared with the control, whereas supplementation with 8 µg had no significant effect (+1.3 percentage points, *p* = 0.650).

When the analysis was restricted to herd A, females receiving semen supplemented with 4 µg of buserelin acetate had a numerically higher farrowing rate (95.0%) than did those in the control group (89.3%, *p* = 0.062) and the 8-µg group (88.2%, *p* = 0.050). Neither the total number of piglets born nor the number born alive differed significantly among these three groups. The total numbers born were 14.4 ± 0.4, 13.1 ± 1.3, and 14.2 ± 0.5 piglets per litter in the control, 4-µg, and 8-µg groups, respectively (*p* = 0.638). The corresponding numbers born alive were 12.7 ± 0.4, 12.1 ± 1.2, and 13.0 ± 0.5 piglets per litter (*p* = 0.797).

## 4. Discussion

The present study demonstrated that supplementation of the first AI semen dose with buserelin acetate was associated with a higher farrowing rate in Landrace × Yorkshire females under tropical commercial conditions. Across treatment doses, the farrowing rate was 90.1%, compared with 86.9% in controls—an increase of 3.2 percentage points. This finding has practical relevance because heat stress has been associated with reduced farrowing rates and increased early embryonic loss in commercial swine herds [7,16,17]. However, the present results do not establish whether supplementation specifically mitigated the effects of heat stress.

These findings extend previous research on buserelin supplementation of boar semen. Taechamaeteekul et al. [14] evaluated doses of 5 and 10 µg at the first insemination in 231 Landrace × Yorkshire gilts. Although farrowing rates did not differ significantly among groups, gilts receiving 10 µg produced more total-born and live-born piglets and had a greater litter birth weight than controls. A subsequent study involving 741 gilts and sows found that supplementation with 5 µg increased the total number born, the number born alive, and litter birth weight in gilts, but yielded no significant reproductive benefits in sows; overall conception and farrowing rates were also unaffected [15]. In contrast, the principal improvement in the present study was in farrowing rate, whereas litter size did not differ significantly between groups. The numerical increase of 0.9 piglets per litter among treated gilts was nevertheless consistent with the direction of previously reported response [15]. Differences among studies may reflect sample size, female reproductive status, herd management, insemination schedules, dose, and baseline reproductive performance. With 2780 females across five herds, the present study represents a broader range of commercial conditions, although responses may still depend on the population and management practices.

A plausible explanation for the improvement in farrowing rate is better alignment between insemination and ovulation. Buserelin is a GnRH agonist that stimulates pituitary LH release and promotes ovulation in females with responsive preovulatory follicles. Injectable buserelin has been shown to induce an LH surge and synchronize ovulation in weaned sows [18]. Under tropical conditions, GnRH administration at estrus onset shortened the estrus-to-ovulation interval from 63.6 ± 9.6 to 37.5 ± 3.3 h [6]. Similarly, intramuscular administration of 10 µg of buserelin at 72 h after weaning reduced this interval from 64.2 to 38.3 h, although only 58% of treated sows ovulated within the expected 32–56 h period. The proportion was lower during the hot season than during the cool season (48% vs. 75%), indicating variation in treatment responsiveness [11]. These findings support the physiological rationale for improving insemination–ovulation synchrony but cannot be directly extrapolated to administration through semen.

For buserelin delivered in extended semen, the proposed endocrine pathway involves absorption across the reproductive tract mucosa followed by stimulation of LH release. However, sufficient absorption to induce an ovulatory response has not been demonstrated under the conditions of the present study. Previous research also reported improved pregnancy and farrowing rates after supplementation of the first insemination dose, without directly establishing an effect on ovulation timing [13]. If this route reduced variation in ovulation timing, it could increase the likelihood that viable sperm were present when oocytes became available. Nevertheless, direct effects on sperm function or uterine contractility should not be assumed.

The higher farrowing rate without a significant increase in litter size is compatible with an improvement in the proportion of females establishing and maintaining pregnancy, without a detectable change in litter size among those that farrowed. Farrowing rate alone, however, cannot distinguish improved fertilization from reduced pregnancy loss, and the numerical increase in litter size among treated gilts does not establish an effect on ovulation rate or embryo survival. Likewise, significant responses at 4 and 5 µg, but not at 8 µg, do not establish an optimal dose or demonstrate that the higher dose is less effective. Dose allocation differed among herds, and subgroup sizes were unequal, limiting interpretation of a dose–response relationship. Control females also received two or three inseminations, whereas treated females received two; therefore, the observed outcomes reflect the treatment regimens as implemented.

Further studies should characterize buserelin absorption and the associated endocrine and ovarian responses following administration through semen. Measuring circulating buserelin and LH alongside ultrasonographic monitoring of follicular development and ovulation while standardizing insemination schedules would help test the proposed mechanism. Evaluating these responses across reproductive categories and thermal conditions would also help identify females and management circumstances most likely to benefit from supplementation.

### 4.1. Herd Effects

The magnitude of the response to buserelin varied among herds, although a significant improvement in farrowing rate was detected only in Herd C (86.5% vs. 90.1%). Such variation may reflect differences in baseline fertility, sample size, genetics, parity structure, estrus detection, AI timing, semen handling, nutrition, environmental control, and the physiological status of females at insemination. In particular, herds with high baseline farrowing rates may have limited scope for further improvement, creating a ceiling effect. Manjarín et al. [13] similarly reported that adding 2 µg of buserelin to the first semen dose increased farrowing rate from 84.9% to 93.2%, whereas the effects of other seminal additives were less apparent, possibly because of the herd’s generally high reproductive performance and controlled environmental conditions. They further suggested that seminal additives may produce greater benefits when reproductive performance is compromised, such as during periods of seasonal infertility or inadequate environmental control. Therefore, the significant response in Herd C may indicate that buserelin is particularly beneficial when insemination–ovulation synchrony or baseline fertility is suboptimal. However, a significant result in only one herd does not necessarily establish a herd-specific biological effect, particularly when herd sample sizes differ. Further multicenter studies should evaluate treatment-by-herd interactions and determine which management, environmental, and reproductive factors predict favorable responses to seminal buserelin supplementation.

Although buserelin supplementation did not significantly affect litter size within any of the five herds, treated females generally showed favorable numerical responses. Total-born litter size increased by 0.1–0.6 piglets in four of the five herds, and the number born alive increased consistently by 0.2–0.6 piglets across all herds. The absence of statistical significance may reflect the relatively small effect size, variation in litter traits, and reduced statistical power when females were analyzed separately by herd. These modest responses contrast with those of Taechamaeteekul et al. [14], who reported that supplementing the first semen dose with 10 µg of buserelin increased the total number born from 13.2 ± 0.3 to 14.0 ± 0.3 piglets, the number born alive from 12.3 ± 0.3 to 13.2 ± 0.4 piglets, and live-born litter weight from 15.6 ± 0.3 to 17.0 ± 0.4 kg in estrus-synchronized gilts. In contrast, 5 µg did not significantly improve litter size, suggesting that the response may be dose-dependent. The smaller and nonsignificant increases observed in the present study may therefore be related to the lower doses used (4–8 µg), the inclusion of both gilts and sows with heterogeneous follicular and reproductive status, and differences among herds in genetics, management, AI timing, and baseline prolificacy. Nevertheless, the consistent numerical increase in live-born piglets across all herds supports a potential beneficial effect. Improved synchronization between insemination and ovulation may increase the proportion of oocytes fertilized by viable sperm; however, the resulting litter size is also limited by ovulation rate, fertilization capacity, embryonic survival, and uterine capacity. Further dose–response studies stratified by reproductive status and herd conditions are needed to determine whether higher or more precisely timed buserelin supplementation can produce consistent improvements in litter size.

### 4.2. Female Reproductive Status

The response to buserelin appeared to vary according to female reproductive status. The clearest numerical benefit was observed in gilts, in which treatment increased the total number born by 0.9 piglets per litter (12.7 ± 0.4 vs. 11.8 ± 0.4) and the number born alive by 0.7 piglets (11.3 ± 0.4 vs. 10.6 ± 0.4). This tendency agrees with Taechamaeteekul et al. [15], who found that supplementing semen with 5 µg of buserelin significantly increased the total number born (18.8 ± 2.1 vs. 14.2 ± 1.0), the number born alive (17.6 ± 1.9 vs. 13.4 ± 0.9), and litter birth weight (23.8 ± 2.1 vs. 17.4 ± 1.0 kg) in gilts, whereas no significant improvements in litter traits were detected in sows. Gilts may benefit more because they generally exhibit greater variation in follicular development and ovulation timing; therefore, GnRH-induced synchronization may improve the alignment of ovulation with the availability of viable sperm. However, the gilts in the previous study had been synchronized with altrenogest, which may have promoted more uniform follicular development and enhanced their response to buserelin. In contrast, the more modest and nonsignificant responses observed in sows in the present study (+0.3 total-born and +0.4 live-born piglets) may reflect differences in follicular maturity, metabolic status after lactation, and the timing of the first AI relative to estrus and ovulation. No clear response was detected in repeat-service females, but this group was small (*n* = 88) and likely heterogeneous in the causes of previous reproductive failure; therefore, the absence of significance should be interpreted cautiously. Collectively, these findings suggest that reproductive status may modify the response to seminal buserelin, with gilts showing the most consistent potential benefit. Further studies should evaluate treatment-by-status interactions and characterize follicular development, ovulation timing, and the causes of repeat service to identify females most likely to respond.

### 4.3. Seasonal Variation

Although buserelin-treated females consistently produced numerically more total-born and live-born piglets across all seasons, none of the seasonal differences was significant. The largest numerical improvement occurred during the cool season (+0.7 piglets for both traits), compared with relatively small increases during the hot and rainy seasons. This pattern may reflect seasonal variation in the ovarian response to GnRH. Pearodwong et al. [11] reported that only 48% of buserelin-treated sows ovulated within the expected 32–56 h after administration during the hot season, compared with 75% during the cool season. Heat stress may reduce feed intake, increase the mobilization of body reserves, suppress LH pulsatility, and delay follicular growth, resulting in follicles that are less mature and less responsive when buserelin is administered. Furthermore, adverse thermal conditions may impair oocyte quality and embryonic survival, potentially limiting any benefit gained from improved synchronization of insemination and ovulation. Nevertheless, the present numerical pattern does not establish a seasonal treatment effect because all comparisons were nonsignificant, and seasonal responses may also have been confounded by differences in herd, female status, environmental control, or sample size. Further studies should formally evaluate the treatment × season interaction while monitoring temperature–humidity conditions, follicular development, LH release, ovulation timing, body condition, and lactational body-reserve loss to determine whether buserelin is more effective during cooler periods or in heat-stressed females with delayed ovulation.

### 4.4. Future Applications and Research Directions

The present findings have practical implications for commercial swine production. Conventional AI programs use repeated inseminations during estrus to compensate for variation in ovulation timing. Supplementing the first semen dose with buserelin may improve insemination–ovulation synchrony and farrowing success while reducing reliance on accurately predicting ovulation in individual females. This practical, non-invasive approach could therefore support more efficient breeding management under tropical commercial conditions. However, its application requires further refinement before widespread adoption. Future studies should determine the optimal dose and timing of buserelin supplementation and examine its interactions with the insemination schedule, ovulation timing, female reproductive status, parity, environmental conditions, and baseline herd performance. Identifying the females and production conditions most likely to respond will be essential for developing targeted protocols that consistently improve farrowing rate and litter performance.

### 4.5. Study Limitations

Several limitations should be considered when interpreting these findings. First, the 4- and 8-µg buserelin doses were evaluated only in herd A, resulting in relatively small subgroups and limiting the statistical power and generalizability of the dose–response analysis. Second, ovulation timing and endocrine responses were not monitored; therefore, the proposed mechanism underlying the observed fertility improvements could not be confirmed. Third, control females received two or three inseminations depending on the duration of standing estrus, whereas treated females received two, with buserelin administered only at the first AI dose. The treatment regimen was based on the hypothesis that buserelin would induce ovulation within 36–40 h after administration [6] and was intended to avoid insemination after ovulation. Because restricting insemination to two occasions without buserelin supplementation could potentially compromise sow reproductive performance, the control group followed each herd’s conventional insemination protocol. Nevertheless, this difference in insemination frequency limits the ability to distinguish the effects of buserelin from those of the insemination regimen. Finally, although the multicenter commercial design enhanced the practical relevance of the findings, variation in AI practices, herd management, season, and female status may have influenced reproductive outcomes. Further controlled studies with standardized insemination schedules, larger dose-specific groups, and direct assessment of ovulation timing and endocrine responses are warranted.

## 5. Conclusions

In conclusion, supplementing extended boar semen with 4–8 µg of buserelin acetate at the first AI increased the overall farrowing rate of Landrace × Yorkshire females by 3.2 percentage points under tropical commercial conditions. Although litter size was not significantly affected, treated females generally produced numerically more total-born and live-born piglets, with the clearest tendency observed in gilts. This practical and non-invasive method may therefore provide an alternative to intramuscular hormone administration for improving farrowing success in commercial herds. Further studies should clarify the endocrine and reproductive mechanisms involved, determine the optimal dose and insemination schedule, and identify the female characteristics and herd conditions associated with the greatest response to buserelin supplementation.

## Figures and Tables

**Table 1:** Descriptive statistics of reproductive performance in Landrace × Yorkshire crossbred females under tropical conditions (2780 inseminated females and 2459 litters).

Variables	Means ± SD	Range
Parity numbers at insemination	3.0 ± 2.1	0–12
Parity number at farrowing	4.0 ± 2.1	1–13
Farrowing rate (%)	88.5	–
Total number of piglets born per litter	14.1 ± 3.2	2–27
Number of piglets born alive per litter	12.9 ± 3.4	0–25
Stillborn piglets (%)	4.7	–
Mummified fetuses (%)	3.6	–

**Table 2:** Reproductive performance of Landrace × Yorkshire crossbred females from five commercial swine herds (A–E) under tropical conditions (least-squares means ± SEM).

Variables	A	B	C	D	E
Number of sows	748	163	1419	80	370
Parity at service ^1^	2.4 ± 1.9	1.6 ± 0.5	3.0 ± 2.2	4.7 ± 1.4	3.2 ± 1.9
Parity at farrowing ^1^	4.0 ± 1.9	2.6 ± 0.5	4.0 ± 2.2	5.7 ± 1.4	4.2 ± 1.9
Farrowing rate (%)	90.0 ^a^	84.5 ^a^	88.3 ^a^	90.0 ^a^	87.3 ^a^
Total born (piglets/litter)	14.8 ± 0.3 ^a^	12.4 ± 0.4 ^b^	13.9 ± 0.3 ^c^	12.4 ± 0.5 ^b^	12.8 ± 0.3 ^b^
Born alive (piglets/litter)	13.1 ± 0.3 ^a^	10.4 ± 0.4 ^b^	12.7 ± 0.3 ^a^	11.5 ± 0.5 ^b^	11.8 ± 0.3 ^b^
Stillbirth (%)	8.0 ± 0.9 ^a^	4.8 ± 1.2 ^bc^	4.6 ± 0.8 ^b^	3.7 ± 1.3 ^bc^	2.9 ± 0.9 ^c^
Mummy (%)	3.3 ± 1.1 ^a^	10.2 ± 1.4 ^b^	3.4 ± 1.0 ^a^	2.9 ± 1.7 ^a^	4.8 ± 1.1 ^a^

^a,b,c^ Values with different superscripts within the same row differ significantly (*p* < 0.05). ^1^ Data are presented as means ± SD.

## Data Availability

The data presented in this study are available upon request from the corresponding author.
